# A Serum Metabolic Profiling Analysis During the Formation of Fatty Liver in Landes Geese via GC-TOF/MS

**DOI:** 10.3389/fphys.2020.581699

**Published:** 2020-12-14

**Authors:** Yujie Gong, Wentao Lyu, Xingfen Shi, Xiaoting Zou, Lizhi Lu, Hua Yang, Yingping Xiao

**Affiliations:** ^1^Key Laboratory of Molecular Animal Nutrition of Ministry of Education, Key Laboratory of Animal Feed and Nutrition of Zhejiang Province, College of Animal Sciences, Zhejiang University, Hangzhou, China; ^2^State Key Laboratory for Managing Biotic and Chemical Threats to the Quality and Safety of Agro-products, Institute of Quality and Standard for Agro-products, Zhejiang Academy of Agricultural Sciences, Hangzhou, China; ^3^Zhejiang Institute of Veterinary Drug and Feed Control, Hangzhou, China; ^4^Institute of Animal Husbandry and Veterinary Science, Zhejiang Academy of Agricultural Sciences, Hangzhou, China

**Keywords:** overfeeding, fatty liver, serum metabolomic, serum enzymatic activities, serum lipid levels, Landes geese

## Abstract

During the process of fatty liver production by overfeeding, the levels of endogenous metabolites in the serum of geese would change dramatically. This study investigated the effects of overfeeding on serum metabolism of Landes geese and the underlying mechanisms using a metabolomics approach. Sixty Landes geese of the same age were randomly divided into the following three groups with 20 replicates in each group: D0 group (free from gavage); D7 group (overfeeding for 7 days); D25 group (overfeeding for 25 days). At the end of the experiment, 10 geese of similar weight from each group were selected for slaughter and sampling. The results showed that overfeeding significantly increased the body weight and the liver weight of geese. Serum enzymatic activities and serum lipid levels were significantly enhanced following overfeeding. Gas chromatography time-of-flight/mass spectrometry (GC-TOF/MS) was employed to explore the serum metabolic patterns, and to identify potential contributors to the formation of fatty liver and the correlated metabolic pathways. Relative to overfeeding for 7 days, a large number of endogenous molecules in serum of geese overfed for 25 days were altered. Continuous elevated levels of pyruvic acid, alanine, proline and beta-glycerophosphoric acid and reduced lactic acid level were observed in the serum of overfed geese. Pathway exploration found that the most of significantly different metabolites were involved in amino acids, carbohydrate and lipid metabolism. The present study exhibited the efficient capability of Landes geese to produce fatty liver, identified potential biomarkers and disturbed metabolic pathways in liver steatosis. These findings might reveal the underlying mechanisms of fatty liver formation and provide some theoretical basis for the diagnosis and treatment of liver diseases.

## Introduction

The fatty liver in geese, also called foie gras, is looked upon the delicious foods as caviar, black mushroom by the occidental, which has a rich, buttery, and delicate flavor (Tang et al., 2018). Consumers worldwide enjoy it, and there is a huge international market (Zhu et al., 2011). In theory, fatty liver is due to the imbalance of synthesis, secretion, and deposition of triglycerides (TG) by the liver (Mourot et al., 2006). The enhanced TG availability could further disrupt serum biochemical parameters. It was reported that patients with suspected liver damage are initially subjected to liver function tests that include the assessment of alanine transaminase (ALT), aspartate transaminase (AST), and glutamyl transpeptidase (GGT) in serum (Soga et al., 2011). Different from human fatty liver, geese have the strong capability to store fat in the liver, however, geese generally do not suffer liver fibrosis or liver necrosis, and the functional integrity of the hepatocytes is still preserved (Zheng et al., 2015). In poultry production, this special property is used to produce fatty liver through 2–3 weeks overfeeding (Mourot et al., 2006; Lu et al., 2015). In particular, Landes geese are famous among waterfowl for their fatty liver production, and the average liver weight can reach 700–800 g after a period of overfeeding where high amounts of corn are delivered to the birds to induce liver steatosis (Zhao et al., 2007; Wu et al., 2014; Fernandez et al., 2016). For geese, miraculously, severe liver steatosis can spontaneously return to a normal liver without causing any pathological damage (Zheng et al., 2015). Consequently, Landes geese are considered as an ideal model in biomedical research for the fatty livers of humans and animals.

Metabolomics, as an indispensable platform for system biology and precision medicine, aims to investigate relevant mechanisms by analyzing metabolic profiles of cells, tissues, organs, biofluids, or whole organisms (Nicholson and Wilson, 2003; Wishart, 2016; Wu et al., 2018). As such, metabolomics has been widely implemented in physiology, disease, and toxicology research fields (Kaddurah-Daouk et al., 2008). Some reports have demonstrated that blood metabolites could be used as physiological biomarkers, reflecting metabolic dysfunction, health, and performance *in vivo* (Soga et al., 2006; Mamas et al., 2011). The process of foie gras production by overfeeding is usually simply called liver fattening, which can undoubtedly lead to metabolic disorders, especially endogenous metabolites in serum. However, previous studies mainly focused on gene level and confirmed the roles of key genes involved in liver steatosis that regulate diverse functions such as cholesterol, glucose, and lipid metabolism (Esau et al., 2006; Zheng et al., 2015; Zhang et al., 2018). To date, the biochemical changes in serum caused by liver fattening have not been elucidated from the perspective of metabolomics.

In the present study, in order to understand the serum metabolic mechanism of fatty liver formation, we performed the phased observations of fatty liver formation in Landes geese by overfeeding different days. Meanwhile, for the accuracy and thoroughness, gas chromatography-time-of-flight mass spectrometry (GC-TOF/MS) based metabolomics approach was applied to study the global changes in serum metabolite levels of Landes geese overfed for different days, and to identify potential biomarkers and their involved metabolic pathways so as to provide some theoretical basis for the diagnosis and treatment of liver diseases.

## Materials and Methods

The experiment was conducted in accordance with the Chinese Guidelines for Animal Welfare and approved by the Institutional Animal Care and Use Committee of Zhejiang Academy of Agricultural Sciences (Hangzhou, China).

### Experimental Animals and Samples Collection

**FIGURE 1:**
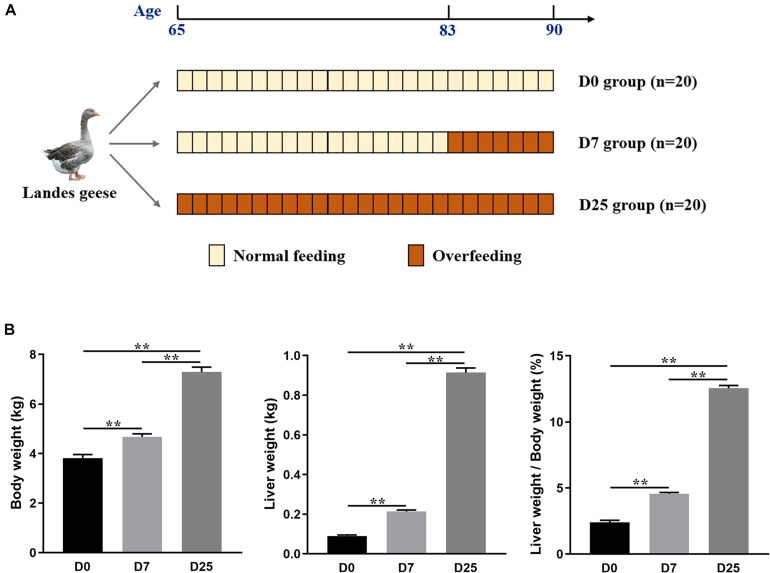
Experimental design used in the study **(A)**. The effects of overfeeding on the body weight, liver weight, and the ratio of liver weight to body weight of Landes geese **(B)**. Values are means with their standard errors. Asterisks indicate statistically significant differences among the groups: **P < 0.01. *n* = 10 per group. D0, overfeeding for 0 day; D7, overfeeding for 7 days; D25, overfeeding for 25 days.

Sixty healthy and of similar weight male Landes geese (65 days old) were selected for this experiment, which was carried out at a farm of ChangXing Glory Goose Industry Co., Ltd. (Huzhou, China). All of these geese grown under natural conditions were fed the same diet. The experimental design is shown in Figure 1A. The geese were randomly divided into one control group (*n* = 20) and two experimental groups (*n* = 20 in each group). The control group (defined: D0 group) was not subjected to gavage, while the geese in two experimental groups were force-fed with a boiled, maize-based diet (five meals of 1,000 g/day per goose) for 7 days (defined: D7 group) and 25 days (defined: D25 group), respectively. At the end of the overfeeding trial, the geese underwent a night fasting and only water was provided. The morning after, 10 geese of similar weight from corresponding group were selected and weighed individually. Subsequently, blood samples were collected in 1.5 mL Eppendorf tubes by puncture of the wing vein, and then centrifuged at 3,000 rpm 4°C for 15 min to separate the serum, which was then stored at −80°C for later analysis. Immediately after blood sampling, the selected geese were slaughtered according to the Administration of Affairs Concerning Experimental Animals of Zhejiang Academy of Agricultural Science. Complete liver stripped from the carcass was weighed and the corresponding value was recorded.

### Detection of Serum Parameters

Serum biochemical parameters, including ALT, AST, GGT, TG, total cholesterol (TC), and high-density lipoprotein cholesterol (HDL), were measured by using routine enzymatic assays with commercial kits (Jiancheng Bioengineering Institute of Nanjing, Nanjing, Jiangsu, China) with the use of an Automatic Biochemistry Analyzer (Hitachi, Tokyo, Japan).

### Sample Preparation for GC-TOF/MS Analysis

One hundred microliter of serum from each sample was mixed with 350 μL of methanol and 20 μL of L-2-chlorophenylalanine (1 mg/mL stock in dH_2_O) in 1.5 mL Eppendorf tube. The mixture was vortexed for 15 s, then centrifuged at 12,000 rpm for 15 min at 4°C to obtain the supernatant. Following, 0.4 mL of supernatant and 60 μL of methoxy amination hydrochloride (20 mg/mL in pyridine) were transferred into the GC/MS glass vial to obtain the extracts, which were dried in a vacuum concentrator at 80°C for 30 min. Subsequently, 80 μL of bis (trimethylsilyl) trifluoroacetamide regent (BSTFA, 1% TMCS, v/v) was added to each sample, and all the samples were incubated for 1.5 h at 70°C. In the process of sample preparation, 10 μL of serum from each sample was pooled as a quality control (QC) sample to evaluate the robustness of the following process. Furthermore, 10 μL standard mixture of fatty acid methyl esters (FAMEs, C8–C16: 1 mg/mL; C18–C24: 0.5 mg/mL in chloroform) was added to the QC sample after cooling it to room temperature. Finally, all samples were thoroughly mixed prior to GC-TOF/MS analysis.

### GC-TOF/MS Analysis

GC-TOF/MS analysis was performed using an Agilent 7890 gas chromatograph system coupled with a Pegasus HT time-of-flight mass spectrometer. The system utilized a DB-5MS capillary column coated with 5% diphenyl cross-linked with 95% dimethylpolysiloxane (30 m × 250 μm inner diameter, 0.25 μm film thickness; J&W Scientific, Folsom, CA, United States). The aliquot (1 μL) of the analyte was injected in the splitless mode. Helium was used as the carrier gas. The front inlet purge flow was 3 mL min^–1^, and the gas flow rate through the column was 1 mL min^–1^. The initial temperature was kept at 50°C for 1 min, then raised to 310°C at a rate of 20°C min^–1^, and finally kept at 310°C for 6 min. The injection, transfer line, and ion source temperatures were 280, 270, and 220°C, respectively. The energy was −70 eV in electron impact mode. The mass spectrometry data were acquired in full-scan mode with the m/z range of 30–600 at a rate of 20 spectra per second after a solvent delay of 6.17 min.

### Statistical Analysis

The GC-TOF/MS raw data were first processed by Chroma TOF 4.3X software of LECO Corporation and LECO-Fiehn Rtx5 database for raw peak exaction, data baseline filtering, and calibration, peak alignment, deconvolution analysis, peak identification, and peak area integration (Kind et al., 2009). The resulting normalized data were analyzed by multivariate statistical analysis using SIMCA software (version 14.1, MKS Data Analytics Solutions, Umea, Sweden), including principal component analysis (PCA) and orthogonal projections to latent structure-discriminate analysis (OPLS-DA). PCA results showed the distribution of the original data. Supervised OPLS-DA was applied to obtain high-level group separation and understand the variables responsible for classification. The significantly different metabolites were identified when the variable importance for the projection (VIP) values > 1.0 in OPLS-DA model and *P* < 0.05 in Student’s *t*-test. In addition, the Kyoto Encyclopedia of Genes and Genomes (KEGG)^1^ was used to search for the related KEGG pathway of the metabolites. MetaboAnalyst^2^, which uses the high-quality KEGG metabolic pathway database as the backend knowledgebase, was used for pathway analysis and visualization.

The experiment data, including the body weight, the liver weight, and the concentrations of serum biochemical parameters, were analyzed using one-way analysis of variance (ANOVA) in SPSS 22.0 software. The significant differences among groups were declared using Tukey’s multiple comparison test. All measures of a statistical significance were found when the probability value was less than 0.05. Results presented in this article are shown as mean ± SEM.

## Results

### Body Weight, Liver Weight, and Blood Index

**FIGURE 2:**
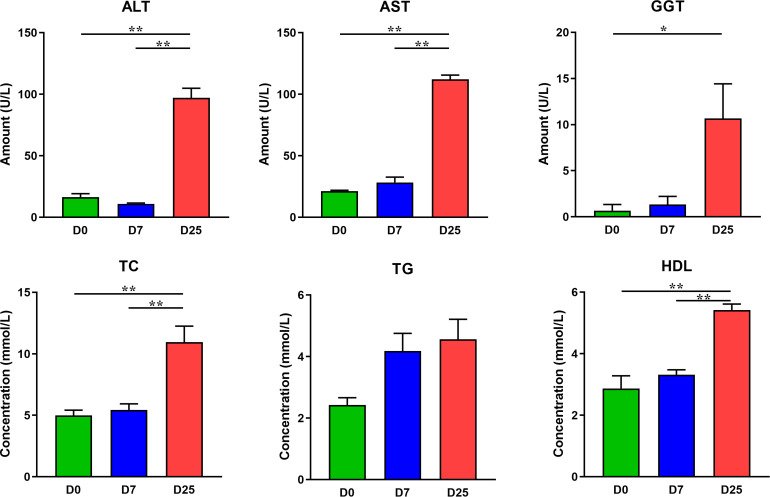
The effects of overfeeding on the serum biochemical parameters of Landes geese. Values are means with their standard errors. Asterisks indicate statistically significant differences among the groups: *P < 0.05, **P < 0.01. *n* = 6 per group. ALT, alanine aminotransferase; AST, aspartate aminotransferase; GGT, γ-glutamyl transpeptidase; TC, total cholesterol; TG, triglycerides; HDL, high-density lipoprotein cholesterol. D0, overfeeding for 0 day; D7, overfeeding for 7 days; D25, overfeeding for 25 days.

After 7 and 25 days of overfeeding, the body weights of overfed geese in the D7 and D25 groups were significantly higher (*P* < 0.01) than that of the D0 group (Figure 1B). The liver weights were considerably higher (*P* < 0.01) of the overfed geese of the D7 and D25 groups and accounted for 4.57 and 12.56% of the body weight, respectively, comparing with 2.40% in the D0 group (Figure 1B). The effects of overfeeding on the blood indexes of Landes geese are summarized in the Figure 2. Relative to the D0 and D7 groups, overfeeding significantly increased (*P* < 0.01) the levels of ALT, AST, TC, and HDL in the serum of the D25 group. After 25 days of overfeeding, the amount of GGT in serum was also increased significantly (*P* < 0.05). With the increase of days for overfeeding, the amount of TG in serum showed an increasing trend, while did not reach a statistically significant level. In addition, no statistically significant difference was found between the D7 and D0 groups in the measured serum biochemical parameters.

### Metabolite Detection and Identification

The GC-TOF/MS platform was applied to study the response of serum metabolic profile to overfeeding. The total ion chromatograms of goose serum samples from the D0, D7, and D25 groups are shown in Supplementary Figure S1. Each peak corresponds to a compound, and the area under the peak represents the relative abundance of the metabolite. In total, 464 peaks were detected by after referring to the LECO-Fiehn Rtx5 database. Further analysis using Chroma TOF 4.3X software to correct the data for missing values, eliminate noise and compliance with an internal standard, 384 valid peaks were retained finally. Among these peaks, 185 compounds were relatively quantified, 126 were marked “analyte” and 73 were labeled “unknown.”

### Metabolic Profiles of GC-TOF/MS Analysis

**FIGURE 3:**
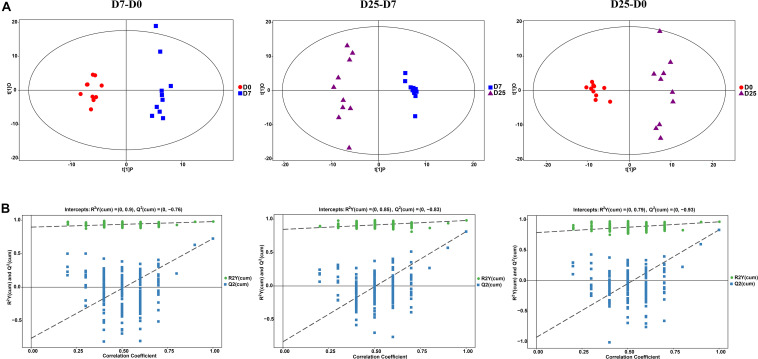
The analysis of GC-TOF/MS of geese serum samples. **(A)** Shows the OPLS-DA score plots among the D0, D7, and D25 groups. **(B)** Shows the OPLS-DA corresponding validation plots among the D0, D7, and D25 groups. GC-TOF/MS, gas chromatography-time-of-flight mass spectrometry; OPLS-DA, orthogonal partial least squares discriminant analysis. D0, overfeeding for 0 day; D7, overfeeding for 7 days; D25, overfeeding for 25 days.

Multivariate statistical analysis methods were implemented to analyze metabolomics data that generate inherent characteristics of GC-TOF/MS. The PCA score plots among the D0, D7, and D25 groups showed the distribution of origin data, which indicated the overall changes in metabolic physiology under the effects of overfeeding. The comparison results of the three groups all showed slight separation (Supplementary Figure S2). To better describe the contribution of overfeeding for classification and higher level of group separation, the OPLS-DA model was used to clarify the different metabolic patterns. The clear separation and discrimination were found in the OPLS-DA score plot for each group comparison (Figure 3A). Further permutation tests were performed to validate the OPLS-DA model. The results of 200 permutation tests showed that the respective R^2^Y and Q^2^ intercept values were 0.90 and −0.76 in the model of the D0 and D7 groups; 0.85 and −0.83 in the model of the D7 and D25 groups; and 0.79 and −0.93 in the model of the D0 and D25 groups (Figure 3B). The low values of the Q^2^ intercept represent that the robustness of the model presents a low risk of overfitting and reliability (Yang et al., 2019). The Q^2^ values are all less than 0 in our tests, thereby indicating that the OPLS-DA model can identify the differences between groups and be utilized in downstream analysis.

### Identification of Significantly Different Metabolites in Goose Serum Samples

**FIGURE 4:**
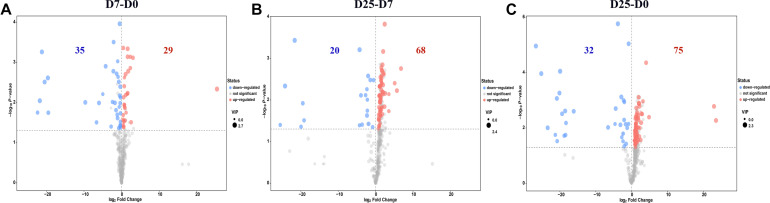
Volcano plots of serum metabolites. **(A)** Shows the D7 group compared to the D0 group. **(B)** Shows the D25 group compared to the D7 group. **(C)** Shows the D25 group compared to the D0 group. The red dots represent metabolites that are up-regulated, the blue dots represent metabolites that are down-regulated, and the gray dots represent metabolites that do not change significantly. The dot sizes indicate the variable importance in the projection (VIP) value. D0, overfeeding for 0 day; D7, overfeeding for 7 days; D25, overfeeding for 25 days.

**FIGURE 5:**
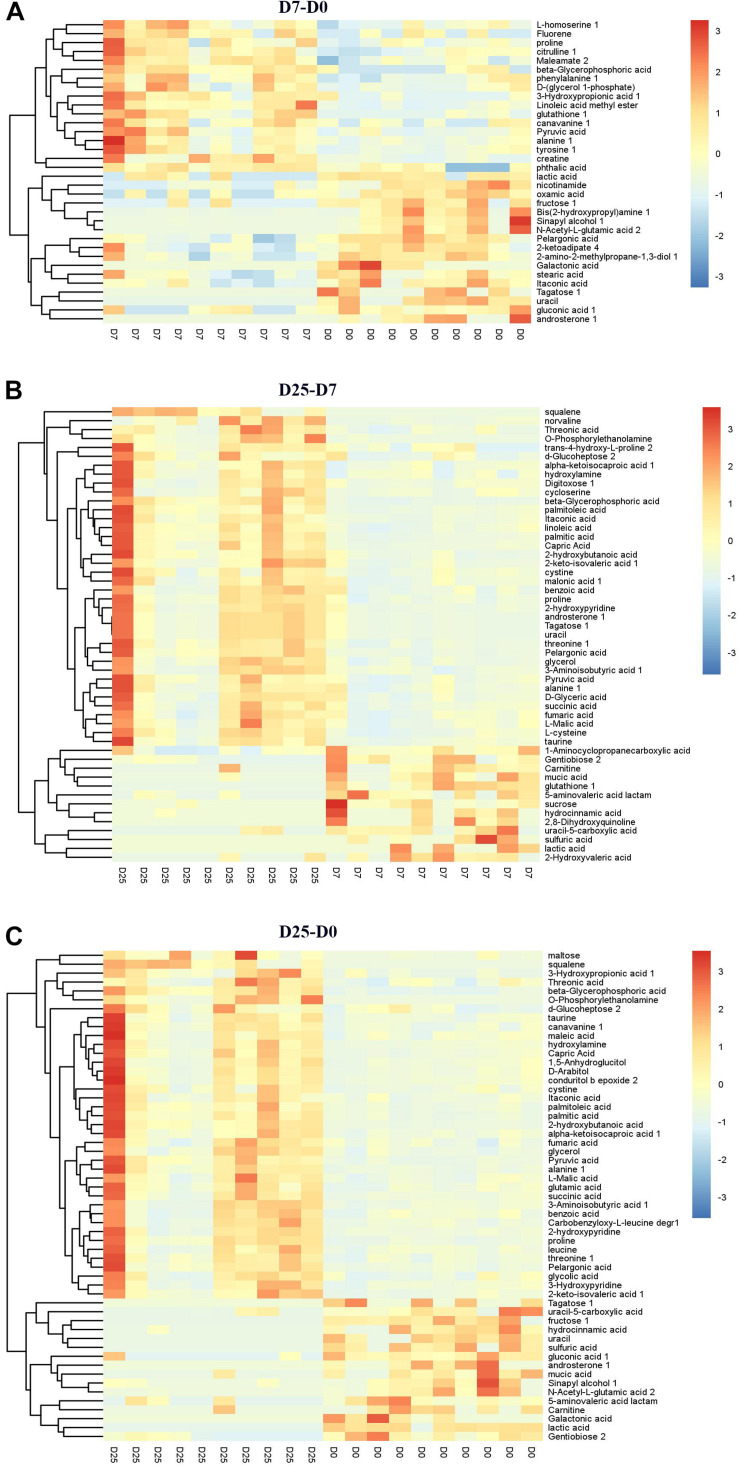
Hierarchical clustering analysis for the significantly different metabolites. **(A)** Shows the D7 group compared to the D0 group. **(B)** Shows the D25 group compared to the D7 group. **(C)** Shows the D25 group compared to the D0 group. The relative metabolite levels are expressed based on the color scale. Red represents upregulation, while blue represents downregulation. D0, overfeeding for 0 day; D7, overfeeding for 7 days; D25, overfeeding for 25 days.

Significantly different metabolites were identified to further confirm significant variables in goose serum. The screening for significantly different metabolites was performed according to the VIP value (VIP > 1.0) and significance test (*P* < 0.05) from the OPLS-DA model. Based on these analyses, 34 significantly different metabolites and 30 significantly different unidentified peaks (named “analyte” or “unknown”) between the D7 and D0 groups, 51 and 37 between the D25 and D7 groups, and 55 and 52 between the D25 and D0 groups are listed in Supplementary Table S1. These screened metabolites and unidentified peaks are shown in the volcano plots (Figure 4). Twenty-nine compounds in the serum were upregulated and 35 compounds were downregulated after 7 days of overfeeding (D7 group vs. D0 group) (Figure 4A). Relative to the D7 group, 68 upregulated compounds and 20 downregulated compounds were detected in the D25 group (Figure 4B). During long periods of overfeeding (D25 group vs. D0 group), undoubtedly, the molecular substances in the serum changed dramatically, with 75 upregulated metabolites and 32 downregulated metabolites (Figure 4C). Subsequently, the distinct characteristics of the significantly different metabolites were displayed in the hierarchical clustering heatmap based on the relative abundance of the identified metabolite (Figure 5). According to the comparison results of the three groups, it can be observed that overfeeding resulted in a continuous increase effect with the number of days in the pyruvic acid, alanine, proline and beta-glycerophosphoric acid. The other two metabolites, 3-hydroxypropionic acid and canavanine, were found to have elevated concentrations after overfeeding for 7 and 25 days (D7 group vs. D0 group; D25 group vs. D0 group), while their concentrations did not change significantly in the comparison between the D25 group and the D7 group. Therefore, these elevated metabolites may serve as potential biomarkers for overfeeding. Furthermore, 25 significantly different metabolites were increased only in the comparison between the D25 group and the D7 group, which are most belonging to organic acids and derivatives (such as succinic acid, succinic acid, 3-aminoisobutyric acid, etc.) or lipids and lipid-like molecules (such as 2-hydroxybutanoic acid, alpha-ketoisocaproic acid, squalene, etc.). On the contrary, a few significantly different metabolites in serum were reduced following overfeeding. Lactic acid is a typical representative of downregulated metabolites, and its changes were particularly obvious. In addition, we found that overfeeding for 7 days resulted in a significant reduction of 7 compounds, including nicotinamide, 2-ketoadipate, oxamic acid, 2-amino-2-methylpropane-1,3-diol, itaconic acid, pelargonic acid, bis(2-hydroxypropyl)amine. These seven compounds (including sulfuric acid, 5-aminovaleric acid lactam, carnitine, hydrocinnamic acid, uracil-5-carboxylic acid, mucic acid, and gentiobiose) showed a downward trend with the increase of overfeeding days (D25 group vs. D7 group).

### Characterization and Functional Analysis of Key Metabolic Pathways

**FIGURE 6:**
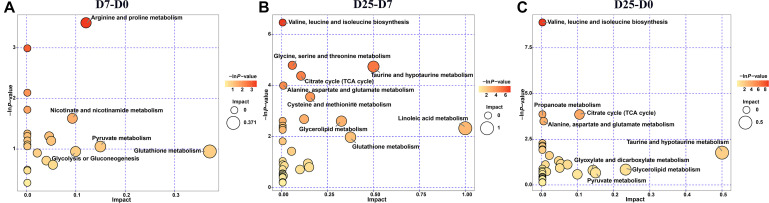
Metabolome view map of significant metabolic pathways. **(A)** Shows the D7 group compared to the D0 group. **(B)** Shows the D25 group compared to the D7 group. **(C)** Shows the D25 group compared to the D0 group. Significantly changed pathways are depicted according to enrichment and topology analysis. Large sizes and dark colors represent high pathway impact and major pathway enrichment, respectively. D0, overfeeding for 0 day; D7, overfeeding for 7 days; D25, overfeeding for 25 days.

To further explore the effects of the significantly different metabolites and identify potential metabolic pathways that respond to overfeeding, we imported the significantly different metabolites into KEGG. The results showed that the numbers of disturbed metabolic pathways in the comparisons of D7 group and D0 group, D25 group and D7 group, and D25 group and D0 group were 22, 32, and 32, respectively. After screening based on the −ln *P*-value and pathway impact scores, the important metabolic pathways are illustrated in a metabolome view map (Figure 6). Five metabolic pathways were enriched in the D7 group relative to the D0 group (Figure 6A). Meanwhile, the D25 group was enriched with nine and eight metabolic pathways compared with the D7 group and the D0 group, respectively (Figures 6B,C). These key metabolic pathways were classified as various amino acids metabolism (valine, leucine, and isoleucine biosynthesis; alanine, aspartate, and glutamate metabolism; glycine, serine, and threonine metabolism; arginine and proline metabolism; cysteine and methionine metabolism; taurine and hypotaurine metabolism; glutathione metabolism), carbohydrate metabolism (citrate cycle; pyruvate metabolism; glycolysis or gluconeogenesis; glyoxylate and dicarboxylate metabolism; propanoate metabolism), lipid metabolism (linoleic acid metabolism; glycerolipid metabolism). As shown in Table 1, several significantly different metabolites (including pyruvic acid, succinic acid, glyceric acid, fumaric acid, linoleic acid, proline, L-cysteine, threonine, glutathione, etc.) were involved in these metabolic pathways, which could be potentially used as biomarkers to liver injure.

**TABLE 1:** Metabolic pathways identified on the significantly different metabolites.

Metabolic pathway	Significantly different metabolites
**D7 group vs. D0 group**	
Arginine and proline metabolism	(1.626) Citrulline^a^ ↑
	(1.566) Proline ↑
	(3.358) Creatine ↑
Nicotinate and nicotinamide metabolism	(0.229) Nicotinamide ↓
Pyruvate metabolism	(1.849) Pyruvic acid ↑
Glycolysis or Gluconeogenesis	(1.849) Pyruvic acid ↑
Glutathione metabolism	(8.101) Glutathione ↑
**D25 group vs. D7 group**	
Valine, leucine and isoleucine biosynthesis	(3.002) Threonine ↑
	(2.414) Pyruvic acid ↑
	(2.639) 2-keto-isovaleric acid ↑
Glycine, serine and threonine metabolism	(1.759) Glyceric acid ↑
	(3.002) Threonine ↑
	(2.864) L-Cysteine ↑
	(2.414) Pyruvic acid ↑
Taurine and hypotaurine metabolism	(2.864) L-Cysteine ↑
	(3.261) Taurine ↑
Citrate cycle (TCA cycle)	(2.144) Succinic acid ↑
	(2.414) Pyruvic acid ↑
	(1.699) Fumaric acid ↑
Alanine, aspartate and glutamate metabolism	(1.699) Fumaric acid ↑
	(2.414) Pyruvic acid ↑
	(2.144) Succinic acid ↑
Cysteine and methionine metabolism	(2.473) Cystine ↑
	(2.864) L-Cysteine ↑
	(2.414) Pyruvic acid ↑
Glycerolipid metabolism	(3.065) Glycerol ↑
	(1.759) Glyceric acid ↑
Linoleic acid metabolism	(3.011) Linoleic acid ↑
Glutathione metabolism	(3.01E–07) Glutathione ↓
	(2.864) L-Cysteine ↑
**D25 group vs. D0 group**	
Valine, leucine and isoleucine biosynthesis	(2.415) Threonine ↑
	(2.029) Leucine ↑
	(4.462) Pyruvic acid ↑
	(3.539) 2-keto-isovaleric acid ↑
Propanoate metabolism	(2.398) Succinic acid ↑
	(7.697) Hydroxypropionic acid
	(6.379) 2-Hydroxybutyric acid ↑
Citrate cycle (TCA cycle)	(2.398) Succinic acid ↑
	(4.462) Pyruvic acid ↑
	(1.955) Fumaric acid ↑
Alanine, aspartate and glutamate metabolism	(1.955) Fumaric acid ↑
	(4.462) Pyruvic acid ↑
	(2.398) Succinic acid ↑
Taurine and hypotaurine metabolism	(2.781) Taurine ↑
Glycerolipid metabolism	(2.601) Glycerol ↑
Glyoxylate and dicarboxylate metabolism	(1.828) Glycolic acid ↑
Pyruvate metabolism	(4.462) Pyruvic acid ↑

**^*a*^The number in the parentheses represents the value of fold change (FC). ↑ and ↓ indicate that the metabolites were upregulated and downregulated in each pair of comparisons.**

## Discussion

What makes geese special is their ability to store energy through overfeeding in the liver to form fatty liver served as a delicious food (Fournier et al., 1997; Davail et al., 2000). In this study, we successfully built a model of goose fatty liver, with the body weight and liver weight significantly increased after overfeeding for 7 and 25 days. In particular, the liver weight of Landes geese overfed for 25 days increased by approximately 800 g, accounting for about 12% of the body weight. This is in accordance with previous results which showed the liver weight increased up to 10-fold after 2 weeks of overfeeding and accounted for up to 10% of the body weight (Hermier et al., 1994). Indeed, our results confirmed the typical characteristic of fatty liver production in Landes geese. Except for geese, ducks are often used to produce fatty livers in the poultry industry. After a period of overfeeding, their liver weight could more than 550 g, approximately eight times its normal weight (Hermier et al., 2003).

In clinical liver diseases such as liver cirrhosis and liver steatosis, the detection of serum enzymatic activities is the most direct method to assess liver damage (Assy et al., 2000; Eslamparast et al., 2014). Serum enzymes mainly come from the liver as the liver steatosis can lead to the hepatocellular inflammation and enzyme synthesis intensification, which will promote the increase of serum enzyme concentration (Zhu et al., 2011). In the present study, overfeeding for 25 days increased the amounts of serum ALT, AST, and GGT, indicating abnormal in liver function of the overfed Landes geese. Similarly, data from published article showed that long-term overfeeding caused liver cell inflammation in Landes geese, accompanied with higher serum enzyme activities (Zhu et al., 2011; Liu et al., 2020). On the other hand, the formation of fatty liver is essentially the disorder of lipid synthesis and secretion, which will inevitably lead to the accumulation of serum lipid (Fournier et al., 1997). Our results showed overfeeding for 25 days caused the significant higher levels of TC and HDL, and a numerical increased TG concentration. These variations in serum lipidemic parameters are consistent with previous studies reporting that overfeeding can induce elevated concentrations of serum lipids in geese (Fournier et al., 1997; Janan et al., 2000). Additionally, we noticed that overfeeding for 7 days did not cause significant variation in serum biological parameters, suggesting that short-term fattening may change the apparent performance, while the body’s metabolism is still in relatively normal operation.

Different aspects of the development of liver steatosis in Landes geese have been studied under experimental conditions (Su et al., 2009; Zheng et al., 2015; Fernandez et al., 2016; Chen et al., 2017). So far, however, the serum metabolic mechanism in this process has not been clarified. Therefore, metabolic profiling with the aid of GC-TOF/MS combined with multivariate statistical analysis was implemented in our study to explore the serum metabolic patterns, and to identify potential contributors to the formation of fatty liver and the correlated metabolic pathways. After 7 days of overfeeding, 34 differentially expressed endogenous metabolites were identified in the serum. These metabolites were involved in 22 metabolic pathways, of which five pathways underwent significant changes. With the increase of days for overfeeding, the number of the altered metabolites in the serum increased gradually. There are 55 differentially expressed metabolites detected in the serum of Landes geese overfed for 25 days, which is much higher than that of the geese overfed for 7 days. Compared with short-term overfeeding, 51 metabolites in the serum of the geese overfed for a long-term changed dramatically. Comprehensive consideration of our research results, metabolites repeated in three comparisons, such as pyruvic acid, alanine, proline, beta-glycerophosphoric acid, 3-hydroxypropionic acid, canavanine, and lactic acid, were selected as potential biomarkers for the study of fatty liver formation. Just as Ma et al. (2017) said in the report, the metabolic pathways identified through the significantly different metabolites represent the typical characteristics response of living systems to pathophysiological stimuli or genetic modification (Kitano, 2002). In the current study, it is noteworthy that several metabolic pathways occur repeatedly in three comparisons. In terms of TCA cycle, it was significantly disturbed whether overfeeding for 7 or 25 days. The disturbed metabolic pathways identified in the current are consistent with classic metabolism and represent the typical features of the dietary or medical intervention on organisms (Rocha et al., 2011; Sun et al., 2016).

In order to systematically demonstrate the metabolic response to overfeeding, the significantly different metabolites combined with corresponding metabolic pathways are shown Table 1. The metabolite with the largest difference between the D0 group and the D7 group was glutathione (8,101-fold higher in the D7 group than that in the D0 group), which is an amino acid and a tripeptide, as well as a well-known antioxidative factor (Ma et al., 2017). This finding is in line with a previous study in human, in which γ-glutamyl dipeptide could serve as biomarkers for discrimination among different forms of liver disease, and had a positive correlation with glutathione, providing specific information for different liver diseases (Soga et al., 2011). The elevated concentrations of citrulline, proline, and creatine, which are products of arginine and proline metabolism, implying the increase of amino acid utilization. Relevant metabolic articles indicated that disturbed metabolism of arginine and proline might play an important role in the obesity progression (Moran-Ramos et al., 2017; Xia et al., 2018), in complete agreement with the increase in body weight and liver weight of Landes geese.

From the dynamic perspective, the changes of serum metabolism in the comparisons of D25 group and D7 group were more similar to the results in the comparisons of D25 group and D0 group. The same metabolic pathways screened for these two comparisons are as follows: valine, leucine, and isoleucine biosynthesis; taurine and hypotaurine metabolism; TCA cycle; alanine, aspartate, and glutamate metabolism; glycerolipid metabolism. The significantly different metabolites enriched in these pathways were pyruvic acid, succinic acid, fumaric acid, 2-keto-isovaleric acid, glyceric acid, threonine, leucine, l-cysteine, taurine, glycerol, which were all up-regulated in the serum of overfed geese. Notably, pyruvic acid could be found in three metabolic pathways, and it also appeared as a key product in pyruvate metabolism, which can be converted into carbohydrates via gluconeogenesis, to fatty acids or energy through acetyl-CoA, and to amino acids, and ethanol (Zeng et al., 2019). The elevated level of pyruvic acid in the serum of overfed geese might be related to the intensification of energy conversion. TCA cycle, glycerolipid metabolism, and pyruvate metabolism are essential metabolic pathways involved in energy supply. As the center of three major nutrients metabolism (carbohydrates, lipids, and amino acids), TCA cycle is the conversion site among sugar, lipid, and amino acid metabolism, and the main way to obtain energy for the body (Owen et al., 2002). The concentrations of pyruvic acid, succinic acid, and fumaric acid enriched in TCA cycle were increased, indicating that overfeeding exerted an important influence on energy metabolism of Landes geese. Other representative metabolites, such as 2-keto-isovaleric acid, threonine, l-cysteine, leucine, are crucial components involved in protein synthesis. The enhanced of protein synthesis may be the main reason for the improvement of apparent growth performance of overfed geese. It has previously been reported that liver fattening in ducks involves the activation of several pathways, including carbohydrate cycling, amino acid metabolism, and lipid synthesis (Hérault et al., 2010; Annabelle et al., 2017), which are consistent with the metabolic pathways identified in our study. On the basis of this research, further study of the metabolic mechanism in the formation of fatty liver should be considered from the perspective of targeted metabolomics in future.

## Conclusion

In summary, our study successfully constructed a model of fatty liver in Landes geese by overfeeding 25 days, which was proved by the increase of body weight, liver weight, blood lipid concentration and serum enzymatic activities. In addition, significantly different metabolites and the metabolic pathways their involved during the formation of fatty liver were identified by comparing the serum metabolome of three groups of geese overfed for different days. The specific effects of these metabolites and the interaction mechanisms of these pathways have not been fully elucidated in the present study. Nevertheless, our results could provide possible directions for future research on the metabolic mechanism in the formation of fatty liver.

## Data Availability Statement

The original contributions presented in the study are included in the article/Supplementary Material, further inquiries can be directed to the corresponding authors.

## Ethics Statement

The animal study was reviewed and approved by the Institutional Animal Care and Use Committee of Zhejiang Academy of Agricultural Sciences (Hangzhou, China). Written informed consent was obtained from the owners for the participation of their animals in this study.

## Author Contributions

YX and HY designed the study. WL and XS conducted the animal trial and the laboratory work. YG conducted a literature review and analyzed the data. YG and YX wrote the manuscript and approved the final version. XZ and LL provided suggestions on the experimental design and the manuscript. All authors contributed to the article and approved the submitted version.

## Conflict of Interest

The authors declare that the research was conducted in the absence of any commercial or financial relationships that could be construed as a potential conflict of interest.

## Footnotes

1 http://www.genome.jp/kegg/

2 http://www.metaboanalyst.ca/

## Supplementary Material

Supplementary Figure 1 Typical GC-TOF/MS chromatograms of goose serum samples from the D0, D7, and D25 groups. The black line represents the D0 group, the red line represents the D7 group and the blue represents the D25 group. D0 group, overfeeding for 0 day; D7 group, overfeeding for 7 days; D25 group, overfeeding for 25 days.

Supplementary Figure 2 PCA score plots among the D0, D7, and D25 groups. D0 group, overfeeding for 0 day; D7 group, overfeeding for 7 days; D25 group, overfeeding for 25 days. PCA, principal component analysis.

Supplementary Table 1 The significantly different metabolites in goose serum samples. ^*a*^The D7 group was compared with the D0 group. ^*b*^The D25 group was compared with the D7 group. ^*c*^The D25 group was compared with the D0 group.

## References

[B1] Annabelle T. Karine R. Marie-Dominique B. Stéphane D. Karine G. (2017). Kinetics of expression of genes involved in glucose metabolism after the last meal in overfed mule ducks. *Mol. Cell. Biochem.* 430 127–137. 10.1007/s11010-017-2960-x 28324238

[B2] Assy N. Kaita K. Mymin D. Levy C. Rosser B. Minuk G. (2000). Fatty infiltration of liver in hyperlipidemic patients. *Dig. Dis. Sci.* 45 1929–1934. 10.1023/a:1005661516165 11117562

[B3] Chen F. Zhang H. Li J. Tian Y. Xu J. Chen L. (2017). Identification of differentially expressed miRNAs in the fatty liver of Landes goose (*Anser anser*). *Sci. Rep.* 7:16296. 10.1038/s41598-017-16632-7 29176640 PMC5701175

[B4] Davail S. Guy G. Andre J. Hermier D. Hoo-Paris R. (2000). Metabolism in two breeds of geese with moderate or large overfeeding induced liver-steatosis. *Comp. Biochem. Physiol. A Mol. Integr. Physiol.* 126 91–99. 10.1016/s1095-6433(00)00190-2 10908856

[B5] Esau C. Davis S. Murray S. F. Yu X. X. Pandey S. K. Pear M. (2006). miR-122 regulation of lipid metabolism revealed by in vivo antisense targeting. *Cell Metab.* 3 87–98. 10.1016/j.cmet.2006.01.005 16459310

[B6] Eslamparast T. Poustchi H. Zamani F. Sharafkhah M. Malekzadeh R. Hekmatdoost A. (2014). Synbiotic supplementation in nonalcoholic fatty liver disease: a randomized, double-blind, placebo-controlled pilot study. *Am. J. Clin. Nutr.* 99 535–542. 10.3945/ajcn.113.068890 24401715

[B7] Fernandez X. Guy G. Laverze J. B. Bonnefont C. Knudsen C. Fortun-Lamothe L. (2016). A kinetic study of the natural induction of liver steatosis in Greylag Landaise geese: the role of hyperphagia. *Animal* 10 1288–1295. 10.1017/S1751731116000161 26915402

[B8] Fournier E. Peresson R. Guy G. Hermier D. (1997). Relationships between storage and secretion of hepatic lipids in two breeds of geese with different susceptibility to liver steatosis. *Poult. Sci.* 76 599–607. 10.1093/ps/76.4.599 9106888

[B9] Gong Y. J. Lyu W. T. Shi X. F. Zou X. T. Lu L. Z. Yang H. (2020). A serum metabolic profiling analysis during the formation of fatty liver in Landes geese via GC-TOF/MS. *Res. Square* 10.21203/rs.3.rs-36620/v1 PMC7767842 33381050

[B10] Hérault F. Saez G. Robert E. Al Mohammad A. Davail S. Chartrin P. (2010). Liver gene expression in relation to hepatic steatosis and lipid secretion in two duck species. *Anim. Genet.* 41 12–20. 10.1111/j.1365-2052.2009.01959.x 19781035

[B11] Hermier D. Guy G. Guillaumin S. Davail S. André J. M. Hoo-Paris R. (2003). Differential channelling of liver lipids in relation to susceptibility to hepatic steatosis in two species of ducks. *Comp. Biochem. Physiol. B Biochem. Mol. Biol.* 135 663–675. 10.1016/s1096-4959(03)00146-5 12892758

[B12] Hermier D. Rousselot-Pailley D. Peresson R. Sellier N. (1994). Influence of orotic acid and estrogen on hepatic lipid storage and secretion in the goose susceptible to liver steatosis. *Biochim. Biophys. Acta* 1211 97–106. 10.1016/0005-2760(94)90143-0 8123687

[B13] Janan J. Bodi L. Agota G. Bardos L. Rudas P. Kozak J. (2000). Relationships between force-feeding and some physiological parameters in geese bred for fatty liver. *Acta Vet. Hung.* 48 89–97. 10.1556/AVet.48.2000.1.10 11402679

[B14] Kaddurah-Daouk R. Kristal B. S. Weinshilboum R. M. (2008). Metabolomics: a global biochemical approach to drug response and disease. *Annu. Rev. Pharmacol. Toxicol.* 48 653–683. 10.1146/annurev.pharmtox.48.113006.094715 18184107

[B15] Kind T. Wohlgemuth G. Lee D. Y. Lu Y. Palazoglu M. Shahbaz S. (2009). FiehnLib: mass spectral and retention index libraries for metabolomics based on quadrupole and time-of-flight gas chromatography/mass spectrometry. *Anal. Chem.* 81 10038–10048. 10.1021/ac9019522 19928838 PMC2805091

[B16] Kitano H. (2002). Systems biology: a brief overview. *Science* 295 1662–1664. 10.1126/science.1069492 11872829

[B17] Liu X. Li P. He C. Qu X. Guo S. (2020). Comparison of overfed Xupu and Landes geese in performance, fatty acid composition, enzymes and gene expression related to lipid metabolism. *Asian-austral J. Anim. Sci.* 33 1957–1964. 10.5713/ajas.19.0842 32054153 PMC7649397

[B18] Lu L. Chen Y. Wang Z. Li X. Chen W. Tao Z. (2015). The goose genome sequence leads to insights into the evolution of waterfowl and susceptibility to fatty liver. *Genome Biol.* 16:89. 10.1186/s13059-015-0652-y 25943208 PMC4419397

[B19] Ma Q. Q. Chen Q. Shen Z. H. Li D. L. Han T. Qin J. G. (2017). The metabolomics responses of Chinese mitten-hand crab (*Eriocheir sinensis*) to different dietary oils. *Aquaculture* 479 188–199. 10.1016/j.aquaculture.2017.05.032

[B20] Mamas M. Dunn W. B. Neyses L. Goodacre R. (2011). The role of metabolites and metabolomics in clinically applicable biomarkers of disease. *Arch. Toxicol.* 85 5–17. 10.1007/s00204-010-0609-6 20953584

[B21] Moran-Ramos S. Ocampo-Medina E. Gutierrez-Aguilar R. Macias-Kauffer L. Villamil-Ramirez H. Lopez-Contreras B. E. (2017). An amino acid signature associated with obesity predicts 2-Year risk of hypertriglyceridemia in school-age children. *Sci. Rep.* 7:5607. 10.1038/s41598-017-05765-4 28717206 PMC5514079

[B22] Mourot J. Guy G. Peiniau P. Hermier D. (2006). Effects of overfeeding on lipid synthesis, transport and storage in two breeds of geese differing in their capacity for fatty liver production. *Anim. Res.* 55 427–442. 10.1051/animres:2006027

[B23] Nicholson J. K. Wilson I. D. (2003). Understanding ‘global’ systems biology: metabonomics and the continuum of metabolism. *Nat. Rev. Drug Discov.* 2 668–676. 10.1038/nrd1157 12904817

[B24] Owen O. E. Kalhan S. C. Hanson R. W. (2002). The key role of anaplerosis and cataplerosis for citric acid cycle function. *J. Biol. Chem.* 277 30409–30412. 10.1074/jbc.R200006200 12087111

[B25] Rocha C. M. Carrola J. Barros A. S. Gil A. M. Goodfellow B. J. Carreira I. M. (2011). Metabolic signatures of lung cancer in biofluids: NMR-based metabonomics of blood plasma. *J. Proteome Res.* 10 4314–4324. 10.1021/pr200550p 21744875

[B26] Soga T. Baran R. Suematsu M. Ueno Y. Ikeda S. Sakurakawa T. (2006). Differential metabolomics reveals ophthalmic acid as an oxidative stress biomarker indicating hepatic glutathione consumption. *J. Biol. Chem.* 281 16768–16776. 10.1074/jbc.M601876200 16608839

[B27] Soga T. Sugimoto M. Honma M. Mori M. Igarashi K. Kashikura K. (2011). Serum metabolomics reveals gamma-glutamyl dipeptides as biomarkers for discrimination among different forms of liver disease. *J. Hepatol.* 55 896–905. 10.1016/j.jhep.2011.01.031 21334394

[B28] Su S. Y. Dodson M. V. Li X. B. Li Q. F. Wang H. W. Xie Z. (2009). The effects of dietary betaine supplementation on fatty liver performance, serum parameters, histological changes, methylation status and the mRNA expression level of Spot14α in Landes goose fatty liver. *Comp. Biochem. Phys. A Mol. Integ. Physiol.* 154 308–314. 10.1016/j.cbpa.2009.05.124 19501665

[B29] Sun H. Wang B. Wang J. Liu H. Liu J. (2016). Biomarker and pathway analyses of urine metabolomics in dairy cows when corn stover replaces alfalfa hay. *J. Anim. Sci. Biotechnol.* 7:49. 10.1186/s40104-016-0107-7 27583137 PMC5006375

[B30] Tang J. Fang Q. Shao R. Shen J. He J. Niu D. (2018). Digital gene-expression profiling analysis of the fatty liver of Landes geese fed different supplemental oils. *Gene* 673 32–45. 10.1016/j.gene.2018.05.122 29879502

[B31] Wishart D. S. (2016). Emerging applications of metabolomics in drug discovery and precision medicine. *Nat. Rev. Drug Discov.* 15 473–484. 10.1038/nrd.2016.32 26965202

[B32] Wu W. Guo X. Zhang L. Hu D. (2014). Association between single nucleotide polymorphisms of fatty acid synthase and fat deposition in the liver of the overfed goose. *Asian-Australas J. Anim. Sci.* 27 1244–1249. 10.5713/ajas.2013.13790 25178366 PMC4150189

[B33] Wu X. Sun H. Xue M. Wang D. Guan L. L. Liu J. (2018). Serum metabolome profiling revealed potential biomarkers for milk protein yield in dairy cows. *J. Proteomics* 184 54–61. 10.1016/j.jprot.2018.06.005 29913267

[B34] Xia B. Zhu Q. Zhao Y. Ge W. Zhao Y. Song Q. (2018). Phthalate exposure and childhood overweight and obesity: urinary metabolomic evidence. *Environ. Int.* 121(Pt 1), 159–168. 10.1016/j.envint.2018.09.001 30208345

[B35] Yang C. Hao R. Du X. Wang Q. Deng Y. Sun R. (2019). Response to different dietary carbohydrate and protein levels of pearl oysters (*Pinctada fucata* martensii) as revealed by GC-TOF/MS-based metabolomics. *Sci. Total Environ.* 650(Pt 2), 2614–2623. 10.1016/j.scitotenv.2018.10.023 30373048

[B36] Zeng Q. Song H. Xu X. Mao W. Xie H. Liang J. (2019). Health effects of kiwi wine on rats: an untargeted metabolic fingerprint study based on GC-MS/TOF. *RSC Adv.* 9 13797–13807. 10.1039/c9ra02138h PMC9063974 35519589

[B37] Zhang J. Wang Q. Zhao X. Wang L. Wang X. Wang J. (2018). MicroRNA-122 targets genes related to goose fatty liver. *Poult. Sci.* 97 643–649. 10.3382/ps/pex307 29182758

[B38] Zhao A. Tang H. Lu S. He R. (2007). Identification of a differentially-expressed gene in fatty liver of overfeeding geese. *Acta Biochim. Biophys. Sin.* 39 649–656. 10.1111/j.1745-7270.2007.00329.x 17805459

[B39] Zheng Y. Jiang S. Zhang Y. Zhang R. Gong D. (2015). Detection of miR-33 expression and the verification of its target genes in the fatty liver of geese. *Int. J. Mol. Sci.* 16 12737–12752. 10.3390/ijms160612737 26057744 PMC4490470

[B40] Zhu L. H. Meng H. Duan X. J. Xu G. Q. Zhang J. Gong D. Q. (2011). Gene expression profile in the liver tissue of geese after overfeeding. *Poult. Sci.* 90 107–117. 10.3382/ps.2009-00616 21177450

