# Supplementary figures and images for: A Serum Metabolic Profiling Analysis During the Formation of Fatty Liver in Landes Geese via GC-TOF/MS
Source: Front Physiol. 2020 Dec 14;11:581699. doi: 10.3389/fphys.2020.581699 (PMC7767842; doi:10.3389/fphys.2020.581699)

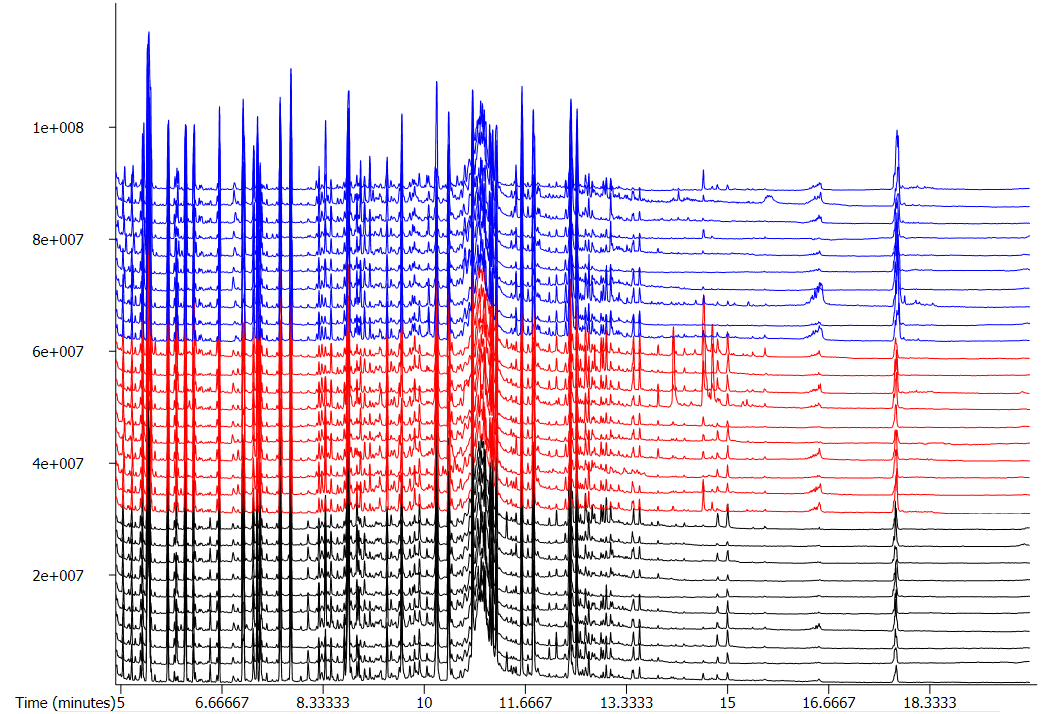

Supplement: Supplementary Figure 1 — Typical GC-TOF/MS chromatograms of goose serum samples from the D0, D7, and D25 groups. The black line represents the D0 group, the red line represents the D7 group and the blue represents the D25 group. D0 group, overfeeding for 0 day; D7 group, overfeeding for 7 days; D25 group, overfeeding for 25 days. [file Image_1.TIF]

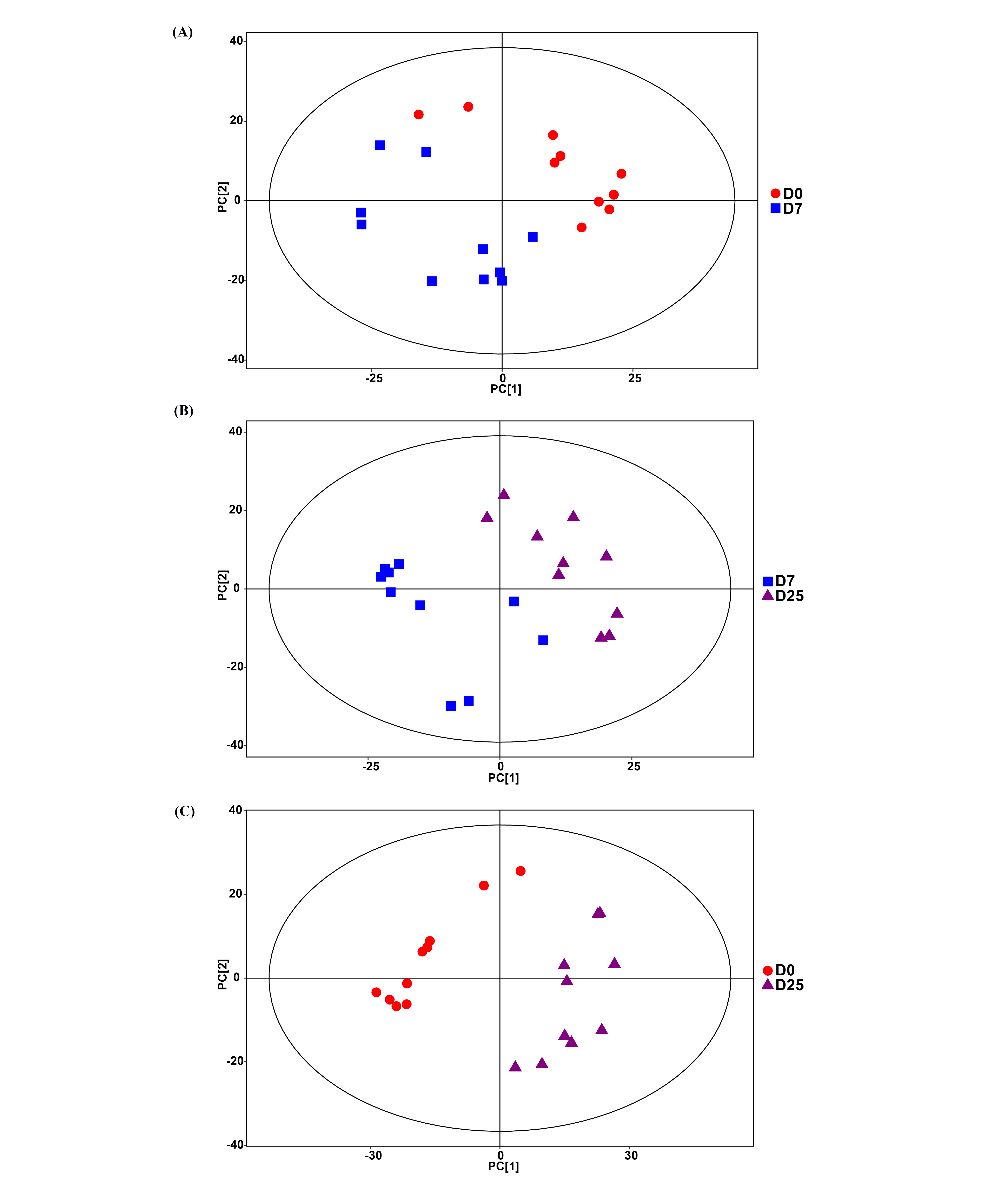

Supplement: Supplementary Figure 2 — PCA score plots among the D0, D7, and D25 groups. D0 group, overfeeding for 0 day; D7 group, overfeeding for 7 days; D25 group, overfeeding for 25 days. PCA, principal component analysis. [file Image_2.TIF]
